# IMB2026791, a Xanthone, Stimulates Cholesterol Efflux by Increasing the Binding of Apolipoprotein A-I to ATP-Binding Cassette Transporter A1

**DOI:** 10.3390/molecules17032833

**Published:** 2012-03-07

**Authors:** Jikai Liu, Zhongbing Zhang, Yanni Xu, Tingting Feng, Wei Jiang, Zhuorong Li, Bin Hong, Zijian Xie, Shuyi Si

**Affiliations:** 1 China Institute of Medical Biotechnology, Chinese Academy of Medical Sciences and Peking Union Medical College, Tiantanxili #1, Beijing 100050, China; Email: mystlumin@yahoo.com.cn (J.L.); okzzb2000@gmail.com (Z.Z.); xuyanni2010@gmail.com (Y.X.); ycttfeng@hotmail.com (T.F.); jiangv@163bj.com (W.J.); l-z-r@263.net (Z.L.); binhong69@hotmail.com (B.H.); 2 Department of Physiology and Pharmacology, College of Medicine, University of Toledo, Toledo, OH 43614, USA

**Keywords:** ABCA1, regulator, high-throughput screening, xanthone, anti-atherosclerosis agent, cholesterol efflux

## Abstract

It is known that the ATP-binding cassette transporter A1 (ABCA1) plays a major role in cholesterol homeostasis and high density lipoprotein (HDL) metabolism. Several laboratories have demonstrated that ABCA1 binding to lipid-poor apolipoprotein A-I (apoA-I) will mediate the assembly of nascent HDL and cellular cholesterol efflux, which suggests a possible receptor-ligand interaction between ABCA1 and apoA-I. In this study, a cell-based-ELISA-like high-throughput screening (HTS) method was developed to identify the synthetic and natural compounds that can regulate binding activity of ABCA1 to apoA-I. The cell-based-ELISA-like high-throughput screen was conducted in a 96-well format using Chinese hamster ovary (CHO) cells stably transfected with ABCA1 pIRE2-EGFP (Enhanced Green Fluorecence Protein) expression vector and the known ABCA1 inhibitor glibenclamide as the antagonist control. From 2,600 compounds, a xanthone compound (IMB 2026791) was selected using this HTS assay, and it was proved as an apoA-I binding agonist to ABCA1 by a flow cytometry assay and western blot analysis. The ^3^H cholesterol efflux assay of IMB2026791 treated ABCA1-CHO cells and PMA induced THP-1 macrophages (human acute monocytic leukemia cell) further confirmed the compound as an accelerator of cholesterol efflux in a dose-dependent manner with an EC_50_ of 25.23 μM.

## 1. Introduction

ABCA1 is a membrane transporter that directly contributes to HDL biogenesis by mediating the cellular efflux of cholesterol and phospholipids to lipid-poor apoA-I [1,2,3]. This membrane protein has been shown to play a rate-limiting role in controlling the efflux of the excess cholesterol from cells [4,5,6,7,8]. Thus, ABCA1 has become a potential target for new drugs aimed at atherosclerosis treatment. There are several aspects about ABCA1 as a potential drug target that researchers are now interested in. First, it is well documented that the cellular ABCA1 protein level is regulated through transcriptional control. Liver X receptors (LXRs) can form obligate heterodimers with retinoid X receptors (RXRs), which both are members of the nuclear receptor superfamily. LXR/RXR heterodimers induce expression of genes (ABCA1) that mediate cholesterol efflux from cells and its ultimate excretion into bile by recognizing specific LXR-response elements consisting of two direct hexanucleotide repeats separated by four nucleotides. [9] However, most of the ABCA1 upregulators are based on the LXR/RXR system or the peroxisome proliferator-activated receptors (PPARs) pathway which results in an extensive pharmacological effect that may not be limited to ABCA1 [9]. Second, some synthetic amphipathic helical peptide mimics of apolipoprotein can mediate lipid efflux via an ABCA1-dependent or ABCA1-independent pathway, and are considered to have antiatherogenic effects [10]. In addition, ceramide and its analogs can stimulate ABCA1-mediated cholesterol efflux to apoA-I by increasing the expression of ABCA1 on the cell surface [11,12]. Furthermore, ABCA1 has been proven to be involved in some signal transduction pathways, which are essential to its function. Stimulation or inhibition by small molecules on one or more signal pathways may interfere with cholesterol transportation [13,14,15,16]. Finally, small molecules such as BLT-4 and glibenclamide can modulate ABCA1-mediated cholesterol efflux by inhibiting its ATPase activity. Thus, a small molecule drug that is able to immediately increase the catalytic activity of ABCA1 on the membrane of the cells may have therapeutic anti-atherosclerotic effects [17].

ApoA-I is the main protein element of high-density lipoprotein. Binding of apoA-I to the extracellular domain of ABCA1 results in the activation of apoA-I lipidation, a key step in reverse cholesterol transport (RCT) process, and is also one of the several proposed mechanisms that protect against atherosclerotic vascular disease [18,19,20]. Although several studies have suggested a molecular interaction between apoA-I and ABCA1 at the cell surface [1,21,22], the role of ABCA1 as a candidate apoA-I receptor is still a matter of debate. Fitzgerald *et al.* believe that apoA-I can interact with ABCA1 directly and promote lipid efflux [21]. Chambenoit *et al.* have shown an interaction between apoA-I and modulated lipid domains in the cell membranes where lipid molecules were meticulously arranged by ABCA1 [1]. Nevertheless, the more apoA-I binds to the ABCA1 proteins on the surface of cells, the more lipids effuse from the cell, an effect which is considered antiatherogenic. In this study a cell-based-ELISA-like HTS method was developed to screen regulators for binding of ABCA1 to apoA-I. Briefly, the human ABCA1 cDNAs were prepared by reverse transcriptase-polymerase chain reaction (RT-PCR) from MRC-5 cell mRNAs. ABCA1 cDNAs were cloned into the pIRES2-EGFP vector for expression, which was transfected into CHO cells. The selection of stable transfected cells which express human ABCA1 was carried out by treatment of the cells with G418 and a positive EGFP fluorescence signal. Anti-apoA-I antibody and horseradish peroxidase–conjugated second antibody were used to detect the apoA-I binding to the cell. Glibenclamide, which inhibits the activity of the ABC superfamily of proteins and apoA-I binding to ABCA1, was used as a control for the optimization and evaluation of the HTS assay for detection in a multi-well plate format. A library of 2,600 compounds was screened using the developed cell-based-ELISA-like assay, and a hit named IMB2026791 with a xanthone structure enhanced apoA-I -ABCA1 binding on the surface of the CHO-ABCA1 cells in a dose-dependent manner. Further cholesterol efflux assay results proved that increased cholesterol was secreted from CHO-ABCA1 cells and phorbol 12-myristate 13-acetate (PMA) induced THP-1cells in a dose-dependent manner when IMB2026791 was added. The effects of IMB2026791 on the viability of A549 (human lung cancer) cell line was tested with an IC_50_ of 301.7 μM. This type of assay platform can be applied to screening a compound library for active compounds with the ability to specifically induce ABCA1-mediated cholesterol efflux to apoA-I. 

## 2. Results and Discussion

### 2.1. Construction of pIRES2-EGFP-ABCA1 and Evaluation of apoA-I-binding Activity of ABCA1

**Figure 1:**
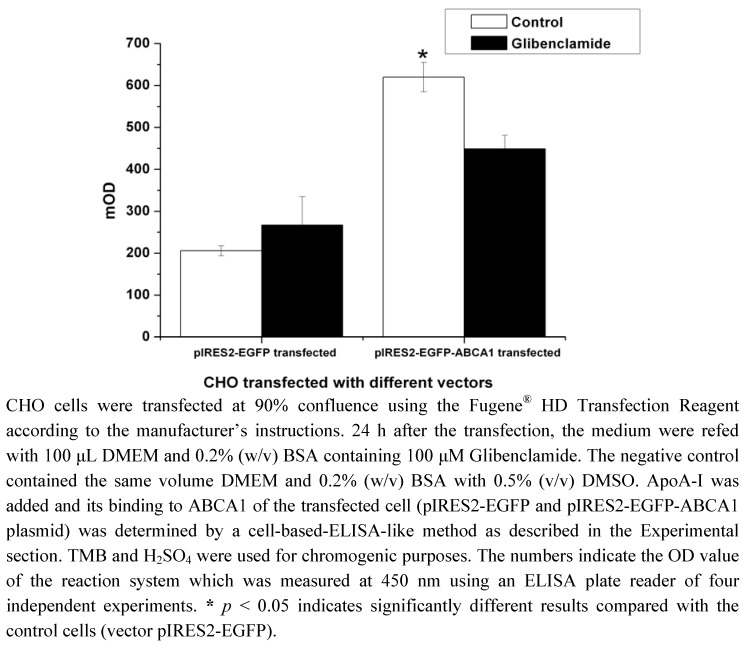
Cell-based-ELISA-like assay of apoA-I binding on the CHO cells transiently transfected with pIRES2-EGFP plasmid or pIRES2-EGFP-ABCA1 plasmid.

The ABCA1 cDNAs were cloned into the pIRES2-EGFP vector for expression. The expression construct, pIRES2-EGFP-ABCA1, was transiently transfected into CHO cells. The apoA-I binding activity was evaluated by a cell-based-ELISA-like assay, and it showed that the amount of apoA-I binding in transfected cells expressing ABCA1 was 3-fold higher than in control cells transfected with blank vector pIRES2-EGFP. There is no significant difference on control cells transfected with blank vector with or without glibenclamide treatments, but an inhibition by glibenclamide was observed on transfected cells expressing ABCA1 (Figure 1, *p* < 0.05).

### 2.2. Cell-Based HTS Assay Optimization

**Figure 2:**
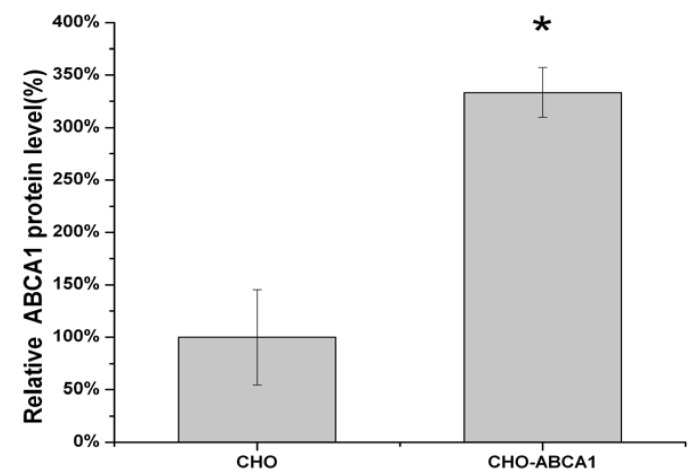
Western blot analyses of ABCA1 protein from pIRES2-EGFP-ABCA1 transfected CHO cells and normal CHO cells. ABCA1 and β-actin antibody were used. The detail was described in the Experimental. Equal quantities of protein (30 µg) were run in each lane. All values represent the mean ± SEM of three independent experiments.

For HTS purposes, stably transfected cell lines expressing high levels of ABCA1 proteins were selected after 20 generations, in which the highest expressing cell line was designated as ABCA1-CHO. Western blot analysis with ABCA1 antibody showed that the stable transfected cell lines produced a 250-KD protein, while CHO cells transfected with vector pIRES2-EGFP as the blank control did not show obvious band (Figure 2).

**Figure 3:**
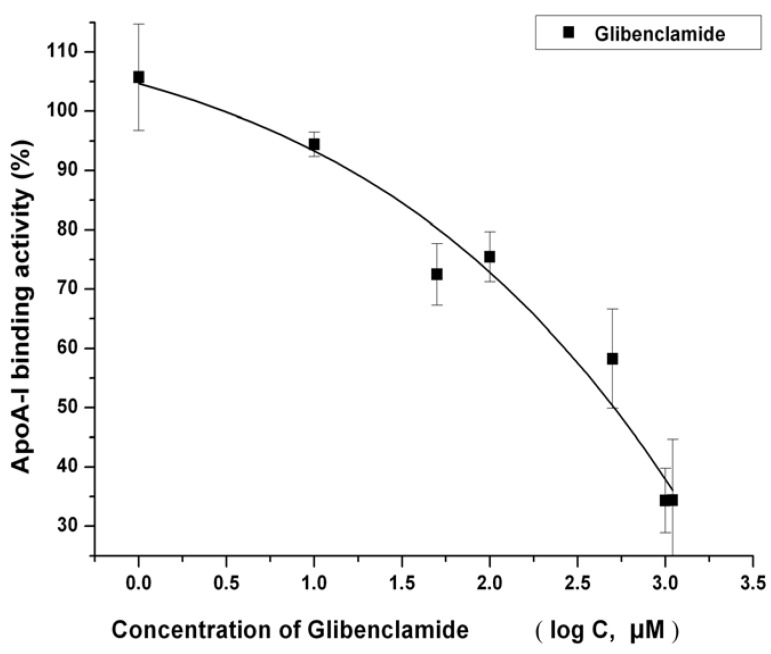
Dose-response curves for glibenclamide. The ABCA1-CHO cells were seeded in 96-well plates at 40,000 cells/well in 200 μL medium B. After 12 h of incubation, the cells at 95% confluence were refed with 100 μL DMEM and 0.2% (w/v) BSA containing the indicated concentrations of negative control and glibenclamide at 37 °C for 1 h. The plate was then incubated at 4 °C for 1 h with or without 10 μg/mL apoA-I and the indicated compounds. The cell-based-ELISA-like assay of apoA-I was performed as described in the Experimental. The signal value of the samples without 10 μg/mL apoA-I was subtracted from the one with 10 μg/mL apoA-I, and the result is the amount of apoA-I bound to the ABCA1. All values represent the mean ± SEM of three independent experiments.

The cells were further characterized by using glibenclamide, a compound which binds tightly and inhibits members of the ABC superfamily including ABCA1. The time of incubation with compound in HTS assay was limited to 2 h in all. Inhibition of ABCA1 by glibenclamide has been reported to occur in the concentration range of 100 to 1,000 μM [23,24]. As shown in Figure 3, the apoA-I binding activity was inhibited by glibenclamide in a dose-dependent manner. It notable that the apoA-I binding activitybegin with 106% in the figure is actually a solvent (DMSO) control data compared with nothing but medium and apoA-I.

During the development of the screening assay using ABCA1-CHO cells in a 96-well format, optimization was focused on acquiring the maximum signal-to-background (S/B) ratio with glibenclamide, so variations in cell numbers per well were investigated. A high S/B ratio (*p* < 0.01) was obtained for 40,000 cells per well. Since the apoA-I is the cholesterol acceptor, the concentration of apoA-I was also determined at 10 μg/mL in the HTS assay. The bound apoA-I reached the highest level when the concentration of added apoA-I was no less than 6 μg/mL. 

### 2.3. High-Throughput Screening for Regulators of ABCA1 Activity

**Figure 4:**
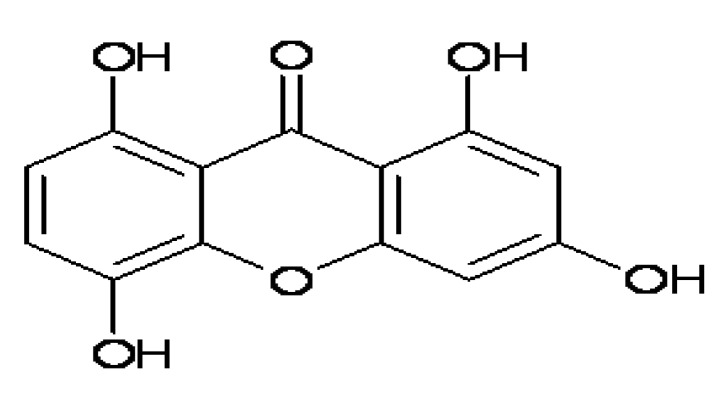
The chemical structure of 1, 3, 5, 8-tetrahydroxyl xanthone (IMB2026791).

**Figure 5:**
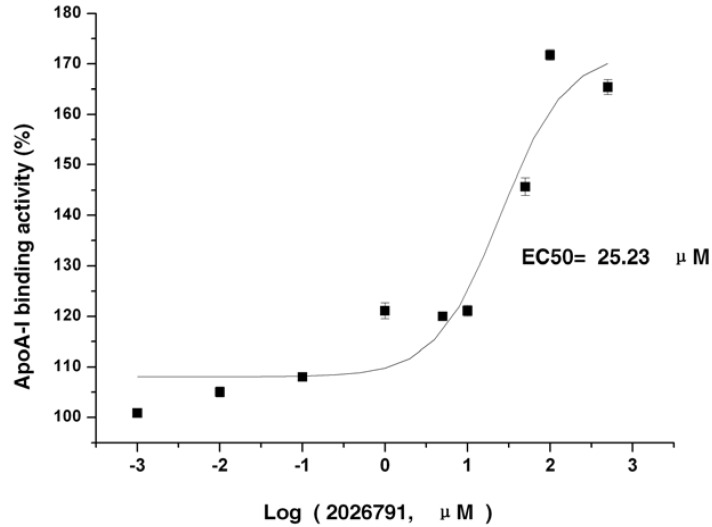
Dose-response curve for IMB2026791. The active compound IMB2026791, identified in the developed HTS, was analyzed at different concentrations for apoA-I’s binding ability to ABCA1. The ABCA1-CHO cells were seeded in 96-well plates at 40,000 cells/well in 200 μL medium B. After 12 h of incubation, the cells at 95% confluence were refed with 100 μL DMEM and 0.2% (w/v) BSA containing the indicated concentrations of negative control and IMB2026791 at 37 °C for 1 h before incubation at 4 °C for 1 h with or without 10 μg/mL apoA-I and the indicated compounds. The cell-based-ELISA-like assay of apoA-I was performed as described in the Experimental. The signal value of the samples without 10 μg/mL apoA-I was subtracted from the one with 10 μg/mL apoA-I, and the result is the amount of apoA-I binding to the ABCA1. All values represent the mean ± SEM of four independent experiments.

A diverse collection of 2,600 compounds were screened at a final concentration of 10 μg/mL as potential ABCA1 activation upregulators. Among these, the compound 1,3,5,8-tetrahydroxyxanthone named IMB2026791 (Figure 4) increased the binding of apoA-I to ABCA1 in a dose-dependent manner as shown in Figure 5.

Conversely, IMB2026791 had no effects on apoA-I binding activity to the control CHO cells which express minimal amounts of ABCA1 (data not shown). However, there were no other hits from the library screen. 

### 2.4. IMB2026791 Enhanced Binding of apoA-I to ABCA1

**Figure 6:**
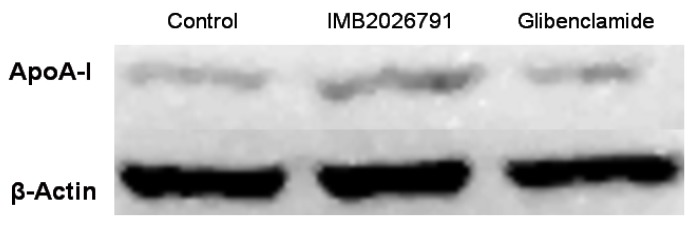
Western blot analysis of apoA-I. Binding of ABCA1-CHO cells was performed after 2 h of incubation with 50 μM IMB2026791 or 100 μM glibenclamide. Equal quantities of protein (30 µg) were run in each lane. A representative immunoblot is shown in the graph, showing the abundance of the immunoreactive apoA-I comparing to β-actin.

**Figure 7:**
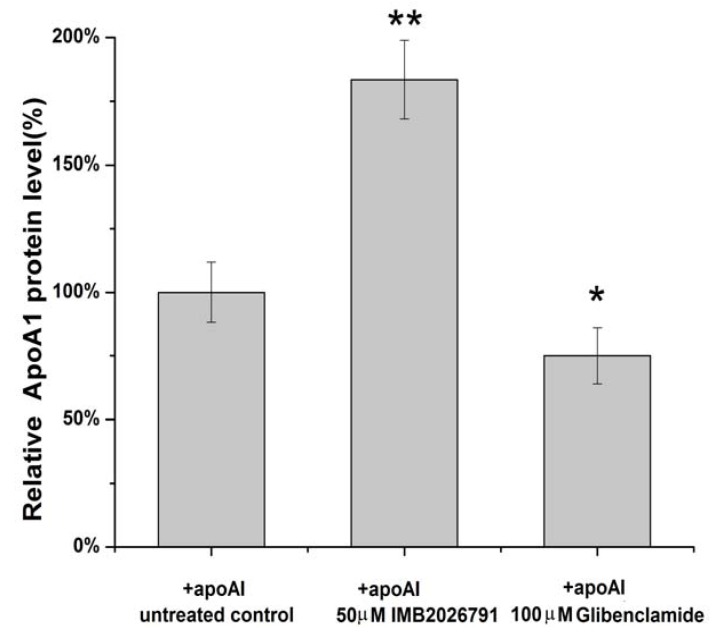
ApoA-I quantified values represent the mean ± SEM of three independent experiments. * *p* < 0.05 and ** *p* < 0.01 indicates significantly different results compared with the control (no compounds).

**Figure 8:**
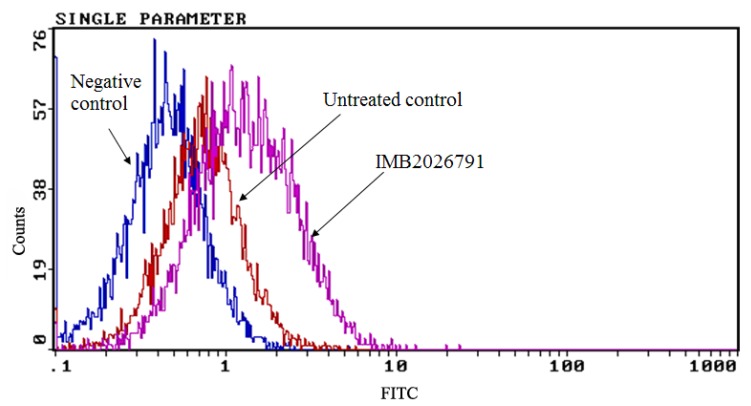
Flow cytometry analysis of the binding ability of apoA-I to the CHO-ABCA1 cells. CHO-ABCA1 cells were treated as described in the Experimental, and the apoA-I that bound to the CHO-ABCA1 cells was measured by flow cytometry with 10,000 cells per sample. Relative fluorescence of each group of cells is demonstrated by an integrogram and presented on log scales. Negative control cells (normal CHO cells) were not treated with any compounds and incubated with apoA-I; untreated control cells (CHO-ABCA1 cells) were not treated with the compounds first or incubated with apoA-I together. IMB2026791 treated ABCA1-CHO cells compared with the control.

**Figure 9:**
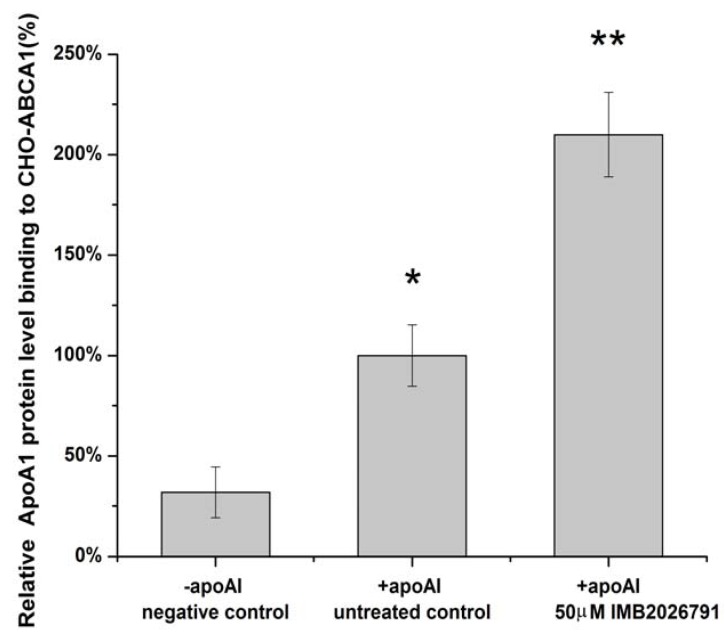
Quantified values represent the mean ± SEM of three independent experiments. * *p* < 0.05 and ** *p* < 0.01 indicates significantly different results compared with the negative control (no compounds and apoA-I).

To confirm IMB2026791 can enhance apoA-I binding to ABCA1, we measured the apoA-I binding of ABCA1-CHO cells by western blot. Figure 6 and Figure 7 show that after 2 h of incubation with 50 μM IMB2026791 or 100 μM glibenclamide. The cells were then incubated at 4 °C for 2 h in the presence of 20 μg/mL apoA-I and the indicated compounds. The cells were then washed with PBS prior to the lysis. Protein extracts from control and treated cells were subjected to SDS-polyacrylamide gel electrophoresis. IMB2026791 enhanced the amount of apoA-I binding of the ABCA1-CHO cells but glibenclamide blocked the binding. The similar results got from flow cytometry displayed in Figure 8 and Figure 9 also show that IMB2026791 enhanced apoA-I binding to the ABCA1-CHO cells**.**

### 2.5. IMB2026791 and Glibenclamide Did Not Change the Surface and Total Expression of ABCA1

**Figure 10:**
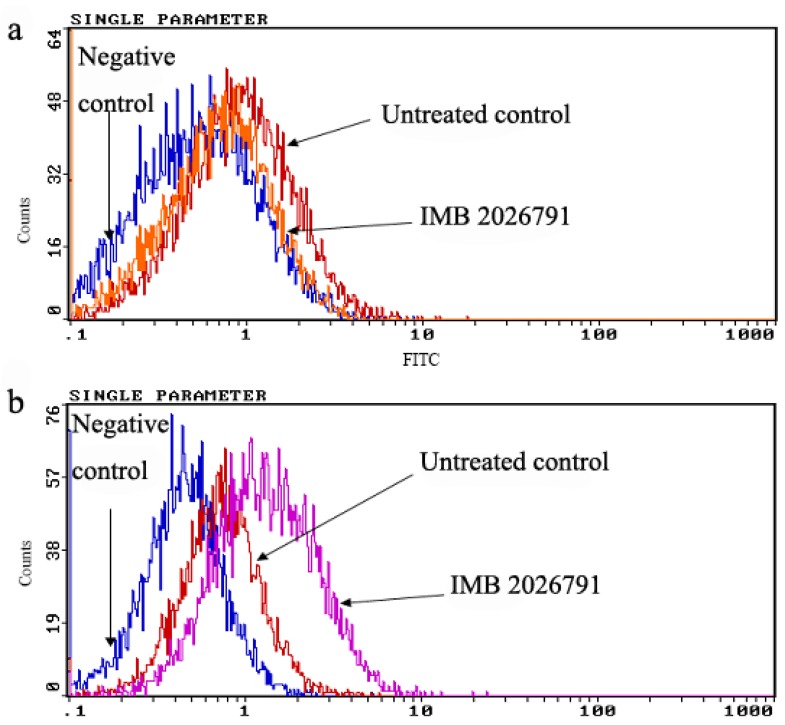
Flow cytometry analysis for the expression level of ABCA1 on the CHO-ABCA1 surface. CHO-ABCA1 cells were treated as described in the Experimental, and the expression of ABCA1 on the CHO-ABCA1 surface was measured by flow cytometry with 10,000 cells per sample. Relative fluorescence of each group of cells are demonstrated by the integrogram and presented on log scales. Negative control cells (normal CHO cells) were not treated with any compounds and incubated with the anti-ABCA1 antibody; untreated control cells (CHO-ABCA1 cells) were not treated with the compounds before being incubated with anti-ABCA1 antibody. (**a**) Glibenclamide treated CHO-ABCA1 cells compared with the control. (**b**) IMB2026791 treated ABCA1-CHO cells compared with the control.

To determine whether IMB2026791 and glibenclamide modulated the surface and total expression of ABCA1, we measured the ABCA1-CHO cell surface expression by flow cytometry and totalexpression by western blot respectively. After 2 h incubation with 50 μM IMB2026791 or 100 μM glibenclamide, no obvious change in expression of ABCA1 was observed on the surface of ABCA1-CHO cells though the amount of apoA-I binding to ABCA1-CHO cells regulated by two compounds separately (Figure 10).

**Figure 11:**
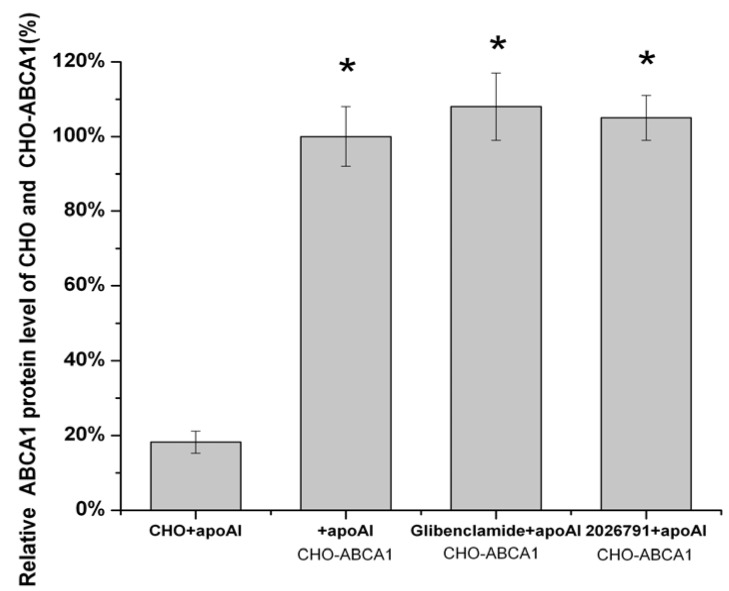
Quantified values represent the mean ± SEM of three independent experiments. * *p* < 0.05 indicates significantly different results compared with the negative control (CHO cells no compounds but apoA-I).

**Figure 12:**
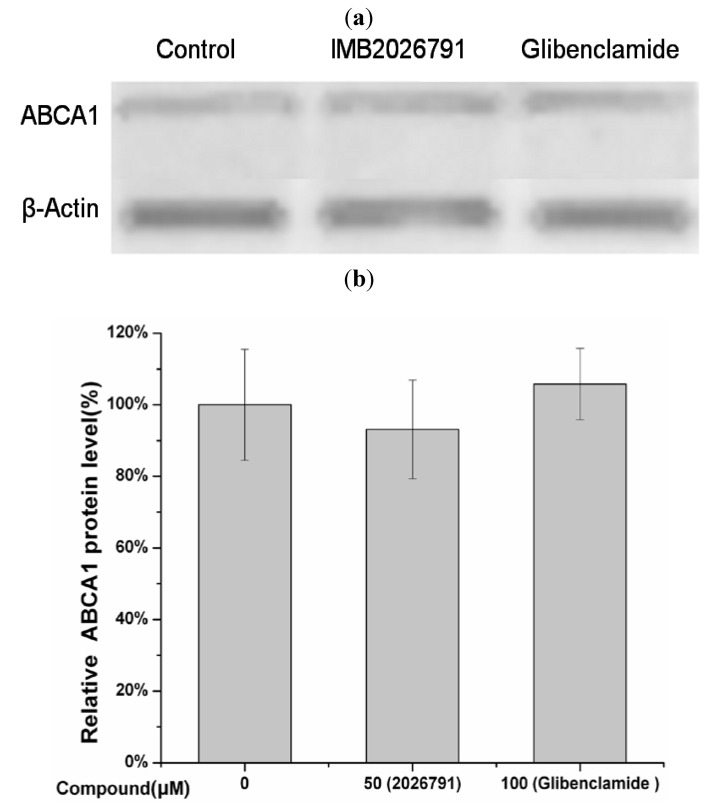
Western blot analysis of protein level of ABCA1 of ABCA1-CHO cells was performed (**a**) after 2 h of incubation with 50 μM IMB2026791 or 100 μM glibenclamide. Equal quantities of protein (30 µg) were run in each lane. A representative immunoblot is shown in the graph, showing the abundance of the immunoreactive ABCA1 comparing to β-actin. Quantified values of (**b**) represent the mean ± SEM of three independent experiments. No significantly different results compared with the control (+apoA-I but no compounds).

A bar graph which show the protein level based on Figure 10 was made (Figure 11). Total expression of ABCA1 in ABCA1-CHO cells show close results got from Western blot by the same treatment of 50 μM IMB2026791 or 100 μM glibenclamide in Figure 12.

### 2.6. Effect of IMB2026791 and Glibenclamide on ABCA1-Mediated [^3^H] Cholesterol Efflux to apoA-I

**Figure 13:**
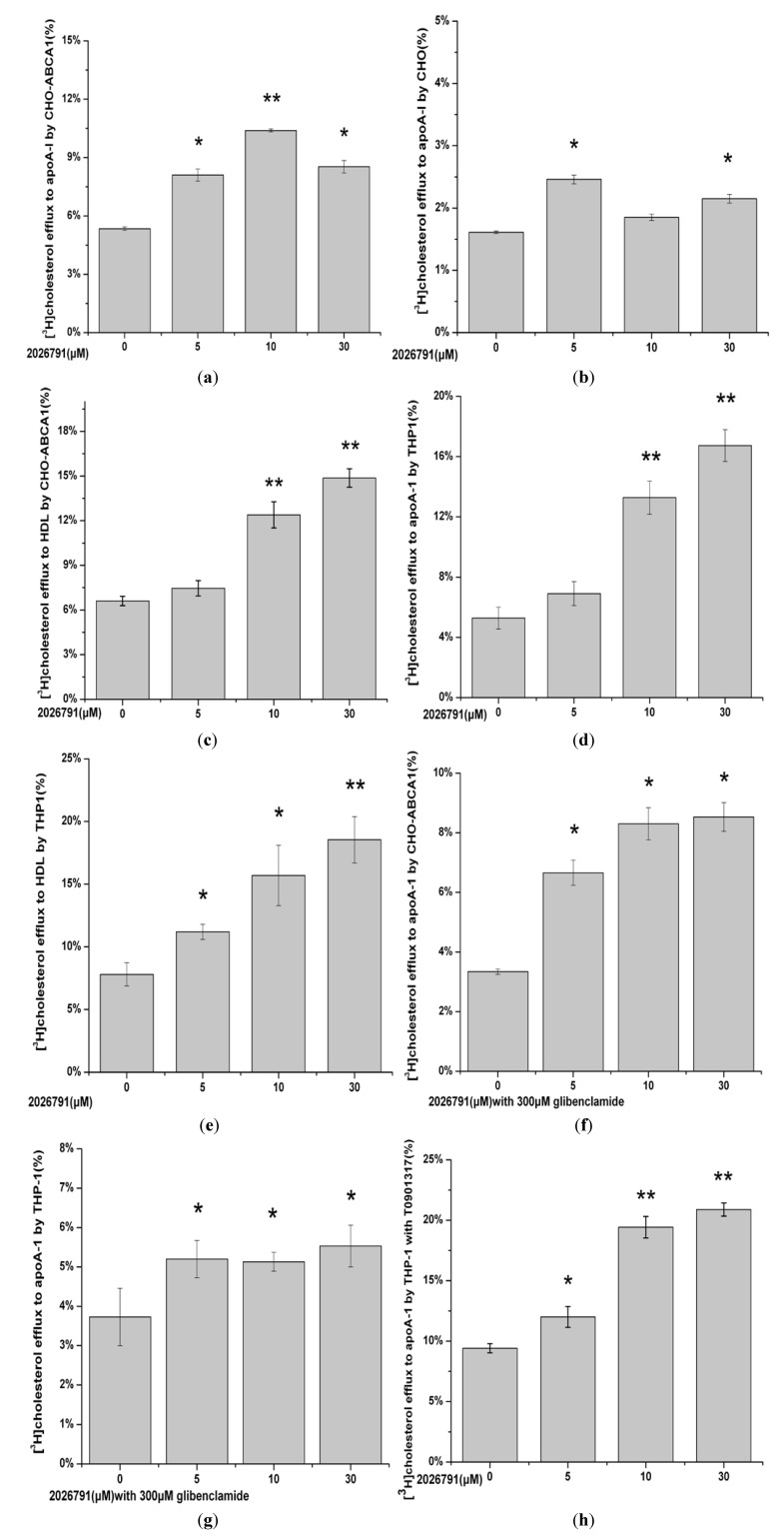
Effect of IMB2026791 on apoA-I-mediated [^3^H] cholesterol efflux in CHO-ABCA1 cells (**a**) and CHO cells (**b**). HDL-mediated [^3^H] cholesterol efflux to CHO-ABCA1 cells (**c**). PMA induced THP-1 macrophages efflux to apoA-I (**d**) and HDL (**e**) was also tested. Glibenclamide in the presence of the xanthone treated CHO-ABCA1 cells (**f**) and PMA induced THP-1 macrophages (**g**) efflux to apoA-I were observed. ApoA-I-mediated [^3^H] cholesterol efflux of THP-1 cells active by LXR agonist T-0901317 was detected (**h**). [^3^H] cholesterol efflux was calculated as described in the Experimental. Cholesterol efflux was defined as the amount of radioactivity in the extracellular media at the end of the 4 h incubation divided by the total amount of cellular radioactivity in the media plus cells which were lysed in 0.1 M NaOH (the percentage efflux). Values represent the means ± standard error of 3 independent experiments. The asterisk denotes a significant difference (* *p* < 0.05 and ** *p* < 0.01).

The enhancement of the binding of apoA-I to ABCA1 by IMB2026791 prompted us to determine whether IMB2026791 will facilitate cholesterol efflux to apoA-I or not. The effect of IMB2026791 on ABCA1-mediated [^3^H] cholesterol efflux from ABCA1-CHO cells to human apoA-I (10 μg protein/mL) were compared (Figure 13a–h). As shown in Figure 13a, 30 μM of IMB2026791 enhanced ABCA1- and apoA-I-dependent cholesterol efflux of ABCA1-CHO cells, while it had little effect on the CHO cells which bear low background efflux in the absence of extra ABCA1 expression. The [^3^H] cholesterol efflux of CHO cells increased, but the efflux was only 1/5 to 1/6 of the ABCA1-CHO cells (Figure 13b). In addition to apoA-I, the effect of the xanthone compound on efflux of ABCA1-CHO cells to human HDL (30 μg protein/mL) were also tested (Figure 13c). More [^3^H] cholesterol efflux to human apoA-1 was observed. Macrophages play central roles in pathogenesis of atherosclerosis through their accumulation of cholesterol. After differentiated with PMA, THP-1 cells behave more like native monocyte-derived macrophages. (23) PMA induced THP-1 macrophages also had significant enhancement of [^3^H] cholesterol efflux to human apoA-I and HDL** (Figure 13**d,e), which increased to more than 3-fold when treated with 30 μM of IMB2026791. The [^3^H] cholesterol efflux of induced THP-1 macrophages to human HDL is more than it to human apoA-I, but not as obvious as to ABCA1-CHO cells. With the presence of 300 μM glibenclamide with different concentration of IMB2026791, inhibited ABCA1- and apoA-I-dependent cholesterol efflux (Figure 13f), compared with IMB2026791 alone. However with the concentration of IMB2026791 increased, the cholesterol efflux has raised slightly. Consistent with findings from previous studies (23-24), apoA-I induced cholesterol efflux to THP-1 cells have been more decreased by glibenclamide than to ABCA1-CHO cells(Figure 13g). The apoA-I-dependent cholesterol efflux of T-0901317 (LXR agonist) activated THP-1 cells was tested. Figure 13h shows a significant increase of cholesterol efflux to apoA-I.

### 2.7. Effects of IMB2026791 on the Viability of Cell Line A549

**Figure 14:**
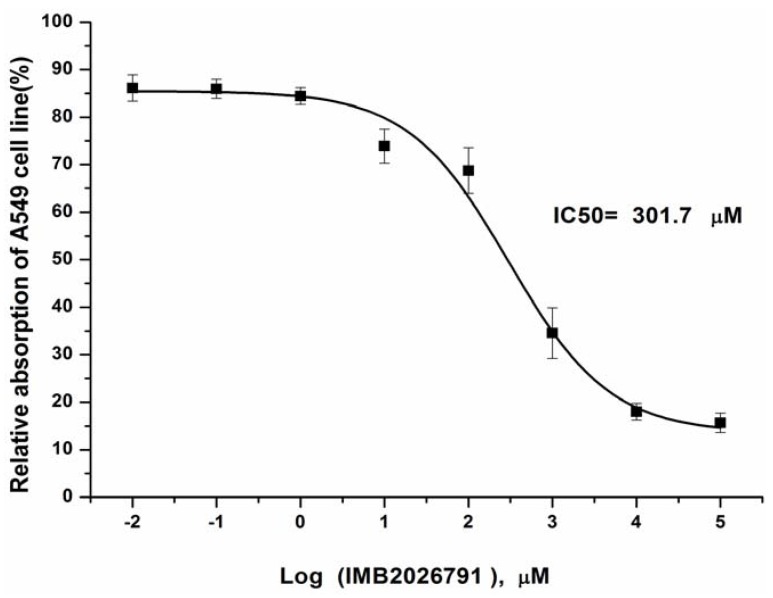
A549 cells were cultured in 96-well plates in full supplement RPMI 1640 medium. After attachment overnight, the medium was replaced and cells were incubated with increasing concentrations of IMB2026791. After treatment, MTT assays were carried out in triplication. The concentration viability curve was fitted and IC_50_ value was determined. Quantified values represent the mean ± SEM of three independent experiments.

The toxicity is critical for its potential as a drug to treat atherosclerosis. The IC_50_ values of IMB2026791 determined by 3-(4,5-dimethylthiazol-2-yl)-2,5-diphenyltetrazolium bromide (MTT) assays against the cell line A549 (31) as shown in Figure 14.

### 2.8. Discussion

In this study, we expressed the ABCA1 in CHO cells using the vector pIRES2-EGFP, which contains the internal ribosome entry site EGFP coding region. Both ABCA1 and the EGFP were translated from a single bicistronic mRNA. Stable transformants was selected by G418 and EGFP. ApoA-I is the main protein in HDL and is considered to be the preferred acceptor for ABCA1-promoted cholesterol and phospholipid efflux. ApoA-I’s EC_50_ value for apoA-I-mediated cholesterol efflux in ABCA1-transfected cells was approximately 2.0 μg/mL [3]. The low EC_50_ indicates a high affinity of ABCA1 for apoA-I. Functional ABCA1 is a receptor for apoA-I binding to the cell surface. Two compounds, glibenclamide and BLT-4, inhibit apoA-I binding to ABCA1, followed by decreasing cholesterol efflux from cells to apoA-I [17]. Enhanced ABCA1 expression by cAMP was accompanied by increased binding of apoA-I to ABCA1 [24]. It was suggested that the increase of cholesterol efflux may be achieved through enhanced apoA-I affinity. In this study, a cell-based-ELISA-like HTS assay targeted at apoA-I binding to cells expressing ABCA1 was established. The method avoids using radioactive isotopes and can be conducted in a 96-well plate format, which is economic and expedient for screening compounds that regulate the activity of ABCA1 and apoA-I-dependent cholesterol efflux. In addition, the time of incubation with compound in HTS assay, western blot and flow cytometry was limited to 2h which preclude an effect of the compound from alterations in gene expression.

A xanthone compound, IMB2026791, was identified and characterized. IMB2026791 not only enhanced the affinity of apoA-I to the cells expressing ABCA1, but also facilitated ABCA1 and apoA-I-dependent cholesterol efflux. The effects of IMB2026791 were verified by Western blot and flow cytometry. Enhanced affinity of HDL to the cells that express ABCA1 also proved the effect of IMB2026791. Previous reports have shown that the putative inhibitor glibenclamide weakens cholesterol efflux and apoE secretion [17,25], which are considered to be atherogenic effects. The results from this study indicate that IMB2026791 facilitated ABCA1-mediated cholesterol efflux, which may be considered an antiatherosclerosis effect. 

Xanthones are a class of polyphenolic compounds that commonly occur in plants and have been shown to have extensive biological and pharmacological activities. Recently, the xanthones and xanthone derivatives were proven to have beneficial effects on some cardiovascular diseases, including ischemic heart disease, atherosclerosis, hypertension and thrombosis which attracted great interest. Another xanthone: 3,4,5,6-tetrahydroxyxanthone with a similar structure to IMB2026791, shows suppression of atherogenesis in apoE deficient mice, which can significantly reduce the area of atherosclerotic lesion, plasma total cholesterol, low density lipoprotein cholesterol (LDL-C) and triglyceride (TG), and increase plasma high density lipoprotein cholesterol (HDL-C) [26]. Mangiferin, a xanthone glucoside, can reduce the plasma total cholesterol, TG and LDL-C levels associated with a significant increase in HDL-C levels and a decrease in the atherogenic index in streptozotocin (STZ) treated diabetic rats indicating its potent antihyperlipidemic and antiatherogenic activities [27]. The protective effects of xanthones in the cardiovascular system may be multiple. In particular, the inhibition of endogenous nitric oxide synthase by xanthones may be the basis for improved endothelial function and for reduction of events associated with atherosclerosis. In this study, enhanced ABCA1 and apoA-I-dependent cholesterol efflux by IMB2026791 may supplement the effect of xanthones on some cardiovascular diseases. 

Although experiments proved that IMB2026791 increased apoA-I binding to ABCA1-CHO cells and promoted the cells’ cholesterol efflux via ABCA1 and apoA-I, the mechanism needs to be further investigated. Using the luciferase HTS assay based on human hepatoma HepG2 cells for screening ABCA1 expression regulators established by Gao *et al.* [28], IMB2026791 had no effect on the transcription level of ABCA1. Analysis by Western blot and flow cytometry determined that no obvious change in the expression of ABCA1 was identified on the surface of ABCA1-CHO cells in the presence of IMB2026791. Thus, two possible mechanisms may explain the effect of IMB2026791 upon ABCA1. One, IMB2026791 may affect the ABCA1 activity through the kinase signal pathways: from cAMP to serine/threonine protein kinases including protein kinase A (PKA), protein kinase C (PKC), and Janus kinase 2 (JAK2) or through Rho family small G-proteins Cdc42 [13,14,15,16]. The other possibility is that IMB2026791 may modulate the ATPase activity of ABCA1 as it is known that the ATPase activity may be consistent with lipid and cholesterol efflux ability. However, the possibility of IMB2026791 affects ABCA1 or apoA-I could not be excluded. Thus, additional studies are necessary to explain how IMB2026791 modulated the activity of ABCA1. Another phenomenon of note is that PMA induced THP-1 cells secrete more cholesterol proportionally than ABCA1-CHO cells. It is reported that PMA induced THP-1 cells express more SR-BI which is also a membrane protein that facilitates the efflux of cholesterol in macrophages [30]. SR-BI may be responsible for the extra observed cholesterol efflux. The effects of IMB2026791 on the viability of A549 cells was also tested, IC_50_ value was determined as 301.7 μM.

## 3. Experimental

### 3.1. Materials

Cell culture media and fetal bovine serum (FBS) were products of Thermo Scientific HyClone (Logan, UT). Human apoA-I (A50620H) was from Biodesign Corporation. Human HDL was from Beijing Union-biology Co., Ltd (Beijing, China), G418, glibenclamide, T0901317, DMSO and PMA were from Sigma. PrimeSTAR HS DNA Polymerase, *Xho* I, *Sac* I, *EcoR* I, *Nhe* I, and *Sal* I were from TaKaRa Biotechnology Co., Ltd (Dalian, China), 1,1′-Dioctadecyl-3,3,3′,3′-tetramethyl-indocarbocyanine perchlorate-labeled human HDL(DiI-HDL) was purchased from Biomedical Technologies (Stoughton, MA, USA), and 96-well polystyrene assay plates were obtained from Costar.

### 3.2. Cell Culture

MRC-5 cells were grown in Minimum Essential modified Eagle’s medium supplemented with 100 U/mL penicillin, 100 U/mL streptomycin (Invitrogen), nonessential amino acids (Invitrogen), and 10% FBS. CHO cells were grown in DMEM with 100 U/mL penicillin, 100 U/mL streptomycin and 10% FBS (medium A). GFP-ABCA1-expressing CHO cells were grown in DMEM with 10% FBS supplemented with 500 μg/mL G418 (medium B). THP-1 cells were grown in RPMI-1640 medium with 10% FBS. All cell cultures were at 37 °C in a humidified 5% CO_2_ atmosphere (cell culture incubator).

### 3.3. Expression Plasmid Construction

Total RNA was prepared from MRC-5 cells using TRIzol reagent (Invitrogen) and reverse transcribed with SuperScript™ III Reverse Transcriptase (Invitrogen). The cDNAs were used as the template for PCR to amplify full length cDNAs consisting of four fragments with the following primers (sense and antisense): 5'-TAGAGCTCAACATGGCTTGTTGGCCTCAG-3' (the underlined nucleotides indicate a *Sac* I site), 5'-ACCCTGATGATTGCCTGCTCCACCAC-3'; 5'-GTTCTGGGCTGGTATTGTGTT-3', 5'-CTGATATTCTCTTCTGGTTGG-3'; 5'-GTTTGACACCTT CCTCTATGG-3', 5'-CATACAGCAAAAGTAGAAGGG-3'; 5'-GTTGTCCCTGCCACACTGGTCA TTA-3', and 5'-AGGTCGACTCATACATAGCTTTCTTTCACTTTC-3' (the underlined nucleotides indicate a *Sal* I site). After sequence verification, the 1831 bp, 1061 bp, 2750 bp and 1624 bp PCR products were digested by *Sac* I and *Xho* I, *Xho* I and *EcoR* I, *EcoR* I and *Nhe* I, *Nhe* I and *Sal* I, respectively. All the digested fragments were ligated and cloned into the pIRES2-EGFP vector (Clontech) using *Sac* I and *Sal* I restriction sites, generating pIRES2-EGFP-ABCA1 containing the full-length ABCA1. The construct was verified by partial DNA sequencing.

### 3.4. Transfection and Selection of Stably Transfected Cell Line

CHO cells were transfected with pIRES2-EGFP-ABCA1 at 90% confluence using the Fugene^®^ HD Transfection Reagent (Roche Applied Science) according to the manufacturer’s instructions. The transfected cells were trypsinized, and then serially diluted with medium A, followed by a 24-h period of incubation. The selection of stably transfected cells was carried out by treatment of the cells with G418 (600 μg/mL) in 96-well culture plates. After 14 days under the selection pressure of G418, single-cell clones with G418 resistance and a positive EGFP fluorescence signal were chosen. A stably transfected clone which with regularly expression level of ABCA1 designated as ABCA1-CHO was maintained in medium B after 20 generations’ culture.

### 3.5. HTS Assay

The compound library used for screening in the present study contained 2,600 chemically diverse, druglike, synthetic or naturally occurring small molecules. Such a platform can be applied to screen for active compounds with the ability to specifically regulate the apoA-I bingding activities to ABCA1 from raw material extracts of plants, marine organisms and fermentation broth of microbes, or a synthetic and natural compound library. Stock compounds were prepared in 96-well plates at 10 mg/mL in 100% DMSO. Compounds occupied 80 wells, and the remaining 16 wells contained DMSO only for negative control purposes. The ABCA1-CHO cells were seeded in 96-well plates at 40,000 cells/well in 200 μL medium B. After 12 h of incubation (when the cells were at 95% confluence), the media was aspirated from the cultures, and the cells were refed with 100 μL DMEM and 0.2% (w/v) fatty acid-free bovine serum albumin (BSA) containing the indicated concentrations of negative control component (DMSO diluted with the medium mentioned above), positive control component (glibenclamide), or screening sample (single wells used for a single compound). After incubation at 37 °C for 1 h, the cells were further incubated at 4 °C for 1 h with the indicated compounds and 10 μg/mL apoA-I. The cells were then washed twice with ice-cold phosphate-buffered saline (PBS)/BSA buffer and fixed with 4% paraformaldehyde for 20 min. After washing twice with PBS, the plate was incubated with blocking buffer: PBS containing 0.1% tween-20 (PBST) with 5% w/v nonfat dry milk at 37 °C for 30 min. The plate was then washed twice with PBS and incubated with human anti-apoA-I antibody (1:1,000, Santa Cruz Biotechnology, Santa Cruz, CA, USA) at 37 °C for 2 h. After washing three times with PBS, the plates were incubated with horseradish peroxidase(HRP)–conjugated goat antirabbit IgG (Santa Cruz) at room temperature for 1 h. The plates were then washed, developed with 100 μL per well of tetramethylbenzidine substrate solution (TIANGEN, Beijing, China) in the dark for 30 min. The reaction was stopped by adding 100 µL of 2 M H_2_SO_4_, and the absorbance was measured at 450 nm using an ELISA plate reader (BMG PolarStar Galaxy, Offenburg, Germany).The HTS assays were performed in duplicate. The S/B ratio was calculated according to the method described by Zhang *et al.* [29] which is 3.5 ± 0.3 and coefficient of variation is 4.6 ± 2.2% under the HTS criteria mentioned above.

### 3.6. Western Blot Analysis

CHO or ABCA1-CHO cells were plated in a 60-mm dish in medium A or medium B at 500,000 cells/dish. After 12 h incubation at 37 °C, the cells were refed with FBS free DMEM containing the compound IMB2026791 or glibenclamide at 37 °C for 2 h. The cells were then incubated at 4 °C for 2 h in the presence of 20 μg/mL apoA-I and the indicated compounds. The cells were then washed twice with ice-cold PBS prior to the lysis in 200 μL/well lysis buffer (50 mM Tris-HCl [pH 7.4], 150 mM NaCl, 1% NP-40, 0.1% SDS). Protein extracts (30 μg) from control and treated cells were subjected to 10% (for apoA-I) or 8% (for ABCA1) SDS-polyacrylamide gel electrophoresis. Proteins were then transferred to a 0.45-μm polyvinylidene difluoride (PVDF) membrane (Millipore, Bedford, MA, USA). Transfer efficiency can be checked by staining proteins on the membrane using Ponceau S (0.5% Ponceau S in 1% acetic acid). Then the membranes were blocked for 1 h at room temperature in PBST with 5% w/v nonfat dry milk. The PVDF membranes were incubated with a rabbit polyclonal antibody against apoA-I or ABCA1 (Santa Cruz Biotechnology) overnight in PBST with 1% BSA, followed by incubation with HRP%#x2013;conjugated goat antirabbit IgG (Santa Cruz Biotechnology) for 1 h at room temperature. An enhanced chemiluminescence (ECL) detection system (Millipore) was used to determine the protein levels. Human β-actin was detected in a similar fashion as an internal control to normalize the intensity of the immunoreactive ABCA1 or apoA-I bands.

### 3.7. Flow Cytometry Analysis

ABCA1-CHO cells were plated in 6-well dishes at 400,000 cells per well in medium B. After 12 h incubation at 37 °C, the cells were refed with FBS free DMEM containing the designated concentrations of compound IMB2026791 or glibenclamide at 37 °C for 1 h. The cells were then incubated at 4 °C for 1 h in the presence of 30 μg/mL apoA-I and the indicated compounds. The cells were washed once with cold PBS, and were then mildly digested with 0.05% trypsin. Cold PBS containing 5% FBS was added to stop the trypsin reaction. The cells were detached from the plate by gentle pipetting and the cell suspension was centrifuged (3 min, 800 ×*g*, 4 °C). The cell pellet was resuspended and washed two times with cold PBS. After fixation with 4% paraformaldehyde for 20 min, the cells were washed twice with PBS and blocked with PBS containing 5% FBS for 30 min. The cells were washed two times with cold PBS, stained with rabbit polyclonal antibody against apoA-I or ABCA1 (Santa Cruz Biotechnology), followed by staining with anti-rabbit IgG-PE (Proteintech Group, Chicago, IL, USA). The cells were analyzed using an Epics XL flow cytometer (Coulter Corporation, Miami, FL, USA).

### 3.8. Cholesterol Efflux Assay

ABCA1-CHO cells and the control CHO cells were plated in 24-well dishes at 200,000 cells per well in medium B and A. After 12 h incubation at 37 °C, the media was removed and the cells were incubated for 24 h in medium B and A supplemented with 0.5 μCi/mL 1, 2[^3^H] cholesterol (Perkin-Elmer). After radiolabeling, the cellular pools of cholesterol were equilibrated by incubation for 24 h in DMEM containing 0.2% bovine serum albumin.

The cells were then washed twice with DMEM and incubated for 1 h in assay medium (DMEM supplemented with 0.2% BSA, 0.5% DMSO, and 25 mM HEPES, pH 7.4). The cells were pretreated with the indicated concentrations of small molecules in the assay medium for 1 h at 37 °C, and then incubated with the same concentrations of compounds in the presence or absence of 10 μg/mL lipid-free recombinant human apoA-I or 30 μg/mL human HDL for an additional 4 h to study the efflux of cellular cholesterol. The supernatant was harvested and clarified by centrifugation (5 min, 6,000 g), the cells were lysed in 0.1 M NaOH, and radioactivity in both fractions was measured by liquid scintillation counting. Efflux medium and cells which were lysed in 0.1 M NaOH (cellular cholesterol) were counted for radioactivity. Cholesterol efflux was represented as the percentage of medium cpm of total cpm (medium + cellular cpm).

THP-1 monocytes were grown in suspension at 37 °C in 5% CO_2_ in RPMI 1640 containing 10% FBS at a cell density around 10^6^/mL. Cells were plated at a density of 680,000 cells per well in 24-well dishes in RPMI 1640 with 10% FBS and 50 ng/mL PMA for 3 days to become fully differentiated macrophages. After radiolabeling, the cellular pools of cholesterol were equilibrated by incubation for 24 h in RPMI 1640 containing 0.2% bovine serum albumin. Differentiated THP-1 macrophages were washed one time with serum-free RPMI 1640 then refed with RPMI 1640 containing 50 ng/mL PMA and 2 μCi/mL 1, 2 [^3^H] cholesterol for 24 h to allow loading of radiolabeled cholesterol. The cells were then washed twice with serum-free RPMI 1640, pretreated with the indicated concentrations of IMB2026791 in RPMI 1640 for 1 hour at 37 °C, and then incubated with the same concentrations of compounds in the presence or absence of 10 μg/mL lipid-free recombinant human apoA-I or 30 μg/mL human HDL for an additional 4 h to allow the efflux of cellular cholesterol. Cholesterol efflux to apoA-1 by differentiated THP-1 macrophages were also test after 12 h’ activation by 10 μM LXR agonist T0901317.The liquid scintillation counting procedure was the same with the ABCA1-CHO cells and the control CHO cells.

### 3.9. Cell Growth Assay

A549 cells were cultured in 96-well tissue culture plates at a cell density of 5,000 cells per well in RPMI 1640 containing 10% fetus bovine serum and 2 mmol/L L-glutamine. After attachment overnight, the medium was replaced and cells were incubated with increasing concentrations of IMB2026791 (ranging from 0.01 μM to 10^5^ μM), resulting in a final volume of 100 μL. After treatment for 48 h, MTT assays were carried out in triplication. The concentration viability curve was fitted and IC50 value was determined.

### 3.10. Data Analysis and Statistical Analysis

Apparent half maximal effective concentration (EC_50_) and the half maximal inhibitory concentration (IC_50_) values were calculated using OriginPro 8.0 (OriginLab, Northampton, MA, USA). All experiments were repeated at least three times, and representative results are presented. Quantitative data are expressed as mean ± SEM. Statistical significance of the data was evaluated by Student’s t-test. Probability values *p* < 0.05 were considered significant. 

## 4. Conclusions

In conclusion, a novel HTS assay for identification of ABCA1 activity upregulators was established. The HDL elevating and atheroprotective functions of ABCA1 have made this transporter an important new target for preventing and even reversing atherosclerotic cardiovascular diseases. A xanthone compound IMB2026791 was identified through this HTS assay and is expected to lead to new therapeutic strategies to prevent atherosclerotic diseases and its complications. Other functions of this transporter and its pathways await future investigations.
